# The Austrian Health Information System “ÖGIS” – supporting implementation of the Public Health Action Cycle in Austria

**DOI:** 10.1007/s43999-022-00012-4

**Published:** 2022-11-24

**Authors:** G. Fülöp, S. Burgmann, G. Maier, S. Mathis-Edenhofer

**Affiliations:** Austrian National Public Health Institute (GÖG), Stubenring 6, A-1010 Vienna, Austria

**Keywords:** Health Data, Health Information System, Epidemiology, Public Health Action Cycle, Austria

## Abstract

The Austrian National Public Health Institute (GÖG) started to develop the Austrian Health Information System (ÖGIS) by combining technology of Geographic Information Systems (GIS) and common approaches of epidemiology in the early 1990ies. Today, ÖGIS is designed as a specific GIS-application, continuously maintained and developed further by in-house development within GÖG and accessible to all employees of GÖG as well as to interested external institutions and persons. ÖGIS covers all major data sources relevant to health, health determinants and the health care system in Austria. It is used for epidemiological research, health reporting, health services monitoring and research as well as for integrated planning of the health care system. After ÖGIS has proven itself as a useful support for implementation of the Public Health Action Cycle in Austria over many years, the maintenance of the ÖGIS databases will be continued and the employment of ÖGIS for purposes of regional health services research will be intensified in the upcoming years.

## Background

In line with the recommendations by the WHO stated in the Environmental Health Action Plan for Europe [1] and the Helsinki Declaration on Action for Environmental Health in Europe [2], the Austrian National Public Health Institute (GÖG) started to develop the Austrian Health Information System (ÖGIS) by combining technology of Geographic Information Systems (GIS) and common approaches of epidemiology (standardized mortality rates, incidence/prevalence rates, significance testing) in the early 1990ies. The roots of this GIS-based concept may be traced back to the 1980ies, when WHO recommended to establish a “Health and Environment Geographic Information System for the European Union” (HEGIS), using standard GIS-packages already available on the market at that time (e.g. ArcInfo, SPANS).

Today, about 30 years later, ÖGIS is designed as a GIS-application, produced and developed further by in-house development within the Austrian National Public Health Institute (GÖG) and accessible to all employees of GÖG. ÖGIS covers all major data sources relevant to health, health determinants and the health care system in Austria. It is used for

- ◦ epidemiological research,
- ◦ health reporting,
- ◦ health services monitoring and research and
- ◦ integrated planning of the health care system.

In early stages of the ÖGIS project, emphasis was put on environmental epidemiology. Over time the focus shifted away from environmental epidemiology which was due to methodological difficulties and due to the seemingly predominant impact of socio-economic conditions on morbidity and mortality in comparison to environmental factors (at least in Austria). In the mid 1990ies it turned out to an increasing extent that the application of a GIS like ÖGIS does not only make sense in the context of epidemiology, health reporting and monitoring, but also in the context of regional health care planning and regional health services research.

By the early 2000ies it was obvious that a health information system like ÖGIS was helpful in literally every aspect of driving forward the implementation of the Public Health Action Cycle ((PHAC) in Austria – including all aspects of this cycle. This is also underlined by the most recent efforts in the fields of health promotion and disease prevention to use ÖGIS via a data-based approach in order to apply tailor-made measures and initiatives per region. In addition, it should be noted that health related geographic information systems in a wider sense have lately received increased attention and have become widely known due to the heavy use of various dashboards designed to visualize infection events in the course of the SARS-CoV-2-pandemic.

## Methods

The basic idea of ÖGIS from the early beginnings was to organize data from all relevant data sources as well as relevant indicators derived from these sources within one common database – thus enabling a synoptic cartographic overview and regional correlation analyses of topics of interest as well (e.g. regional distribution of overweight and smoking habits on the on hand versus prevalence and mortality due to circulatory diseases on the other hand).

**Fig. 1:**
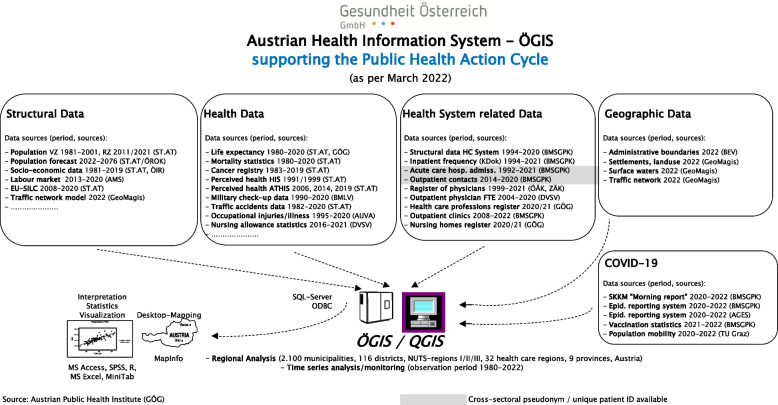
Data sources and data availability in ÖGIS as per 2022

Collecting of the raw data is basically done on the highest available level of spatial resolution (i.e. around 2.100 municipalities in Austria) with observation periods reaching back to the early 1980ies for some of the data sources. Data and indicators are available in ÖGIS on several levels of spatial resolution (ranging from the 9 Austrian provinces to the level of the municipalities). They are organized basically in four data modules (cf. Figure 1):

- ◦ Basic geographic data (administrative boundaries, waters, traffic lines etc.)
- ◦ Structural data (demographic and socio-economic data, degree of education, traffic network models etc.)
- ◦ Health data (life expectancy, mortality and morbidity data, cancer registry, hospital admission statistics, survey data on health behaviour and health status, military medical check-up data, traffic accident data etc.)
- ◦ Health care system related data (availability and utilization of hospitals and nursing homes, supply with general practitioners, specialists in free practice and other health care professions, emergency services etc.)

In early 2020 a special additional module was added in order to provide real time coverage of virtually all aspects of the SARS-CoV-2-pandemic and to support the management of the pandemic in Austria (cf. Figure 1, bottom right). At the same time environmental data and indicators related to the early beginnings of ÖGIS are still available in another special module not directly connected to the GIS (e.g. data on air pollution as to SO2, NO, NO2, O3 and TSP, heat waves, radioactivity, drinking water quality etc.) – they may be of increased importance when it comes to research regarding the consequences of climate change.

One of the core features of ÖGIS are methods to describe and visualise different types of catchment areas, which are derived either from utilisation patterns of health facilities (“actual catchment areas”) or algorithmically using gravity models and/or the principle of the nearest opportunity (“natural catchment areas”, determined via accessibility in road traffic or by public traffic means or via geometric distance).^1^ Furthermore, “natural catchment areas” in combination with small area health statistics features in ÖGIS are also used to depict the local characteristics in the hypothetical catchment area of a preselected location.^2^

Data organization is done via a SQL data base in the background of the GIS, which is continuously improved based on feedback of GÖG employees and of addressees of ÖGIS analysis in the scientific community as well as of policy making groups. Emphasis is put on usability of the system, including minimal response times (real time in most cases) and coherency of the outputs – thus enabling interactive and explorative research, including testing of hypotheses. Data and indicators are basically available on six levels of spatial resolution for almost all data sources in ÖGIS^3^:

- ◦ One State of Austria (around 9 million inhabitants)
- ◦ Nine Provinces (NUTS-II-level according to Eurostat, population ranging from 300.000 to 1.9 million inhabitants)
- ◦ Thirty-five NUTS-III-regions (NUTS-III-level according to Eurostat, population 20.000 to 1.9 million inhabitants)
- ◦ Thirty-two Health care regions (as defined by the “Austrian Health Care Structure Plan—ÖSG”, similar to the NUTS-III-regions, population 50.000 to 800.000 inhabitants)
- ◦ One hundred sixteen Political districts (population 10.000 to 300.000 inhabitants)
- ◦ 2.100 Municipalities (populations ranging from 100 to 300.000 inhabitants)

Environmental data are mostly collected as “spot-related indicators” (e.g. air pollutants concentration at around 180 sites) apart from the ÖGIS SQL database and therefore not directly combinable with indicators related to health and the health care system.

## Results

Queries may be directly sent to ÖGIS and are supported by a user-friendly interface (including drop-down menus, check boxes and quick info). Outputs are displayed via tables, graphics, or maps^4^ (depending on the configuration of the query as defined by the user) and comprise the raw data addressed as well as a number of indicators. Compatibility of ÖGIS outputs to tools for more detailed statistical analysis (e.g. MS-Excel, SPSS, Minitab, R etc.) is ensured.

Data and analyses from ÖGIS are primarily accessible to all employees of GÖG. Additionally, a presentation of maps displaying pre-selected indicators has been placed on the www in order to present some basics regarding epidemiology in Austria to the public (“Regional Health Information System – REGIS”).^5^ This ÖGIS-based presentation will be replaced by a new “Austrian Online-Health-Atlas” (currently under construction) in the upcoming months.

Moreover, users from outside the GÖG may order tailored data and/or indicators, which would be extracted from ÖGIS and sent to the interested persons and institutions (mostly from the scientific community or from providers of health services in Austria and Germany). Provision of data has to comply to the General Data Protection Regulation as defined by the European Union.

**Fig. 2:**
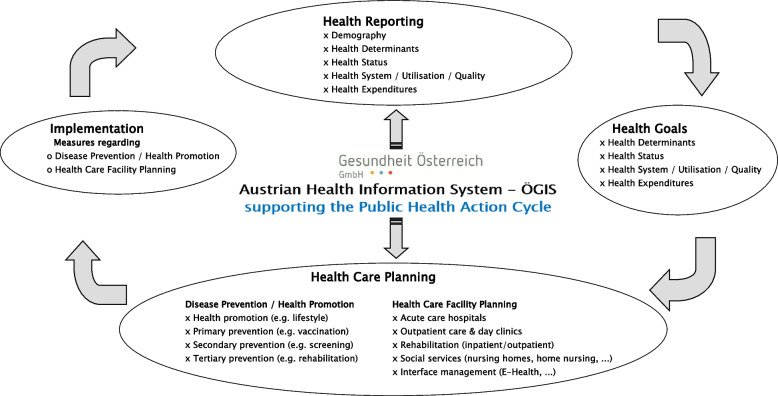
Role of ÖGIS supporting regional health services research and implementation of the PHAC

Examples for employment of ÖGIS data evaluations inside and outside GÖG may be listed along the different sections of the Public Health Action Cycle to be supported by analyses and monitoring tools (cf. Figure 2):

- ◦ Health reporting^6^ and defining health goals^7^—analysis of regional distribution and development over time of health behaviour and of the burden of disease by disease group (including indicator systems for health system monitoring)
- ◦ Health care facility planning: Austrian Healthcare Structure Plan (ÖSG)^8^ as well as nine Regional Healthcare Structure Plans (RSG)^9^ for each of the nine provinces in Austria – background analyses and simulation of planning scenarios
- ◦ Disease prevention and health promotion – identification of hot spots and priority areas for supporting measures in these fields^10^
- ◦ Implementation of measures – support by delivering tailored descriptions, e.g. for defining special features for envisaged primary health care centres according to of regional characteristics of their catchment area^11^
- ◦ Real time management of pandemic crises such as the CoV-pandemic, simultaneously affecting almost all sections of the Public Health Action Cycle virtually at the same time^12^

## Conclusions

ÖGIS was originally developed with a focus on environmental epidemiology. Over years, it has evolved into a universally applicable data tool in the fields of health monitoring, health reporting and health care planning, continuously being adapted to current challenges and needs.

In accordance with the requirements of health monitoring and health care planning (particularly in connection with the Austrian health care reform called “target-based health governance” and started in 2013^13^) databases and features of ÖGIS have been and are being used by a large number of addressees inside and outside the GÖG.

After ÖGIS has proven itself as a useful support for implementation of the Public Health Action Cycle in Austria over many years, the maintenance and updating of the ÖGIS databases will be continued. Furthermore, employment of ÖGIS for purposes of regional health services research will be intensified in the upcoming years.

## Data Availability

Several sources; for further information on the data please contact the corresponding author.

## Footnotes

1 cf. https://www.kbv.de/media/sp/1_F_l_p_Gesundheitsplanung.pdf p. 8–9.

2 cf. https://archpublichealth.biomedcentral.com/track/pdf/10.1186/s13690-022-00821-6.pdf p 4.

3 cf. https://www.zi-congress.de/fileadmin/Abstracts/Vortraege_frei_05.06.2019/P2-2_Fueloep_Gerhard.pdf p. 5–9.

4 cf. https://www.kbv.de/media/sp/1_F_l_p_Gesundheitsplanung.pdf p. 6–10.

5 cf. https://regis.goeg.at/

6 e.g. https://goeg.at/sites/goeg.at/files/2017-06/Gesundheit_und_Krankheit_in_Oesterreich_2004_0.pdf

7 cf. https://gesundheitsziele-oesterreich.at/10-ziele/

8 cf. https://goeg.at/OESG

9 cf. https://goeg.at/RSG_Monitoring

10 cf. https://medonline.at/128987/2014/praeventions-charta-auva/

11 cf. https://archpublichealth.biomedcentral.com/track/pdf/10.1186/s13690-022-00821-6.pdf

12 cf. https://jasmin.goeg.at/1863/1/COVID19_Austria_incidence.mortality.vaccination_vs_socio-economic_aspects.pdf

13 cf. https://broschuerenservice.sozialministerium.at/Home/Download?publicationId=642, p. 10 ff.
